# Systematic review of clinical guidelines for lipid lowering in the secondary prevention of cardiovascular disease events

**DOI:** 10.1136/openhrt-2020-001396

**Published:** 2020-12-18

**Authors:** Rosemary Elisabeth Brown, Paul Welsh, Jennifer Logue

**Affiliations:** 1 BHF Glasgow Cardiovascular Research Centre, Institute of Cardiovascular and Medical Science, University of Glasgow, Glasgow, UK; 2 Lancaster Medical School, Lancaster University, Lancaster, Lancashire, UK

**Keywords:** lipid lowering, general practice, lipids, statins

## Abstract

**Background:**

The WHO recommends that those with established cardiovascular disease should be treated with lipid-lowering therapy, but there is no specific guidance regarding lipid monitoring. Unnecessary general practitioner visits may be a burden for patients and increase healthcare costs. A systematic review of the current guidelines was performed to reveal gaps in the evidence base for optimal lipid monitoring approaches.

**Methods:**

For this systematic review, a search of Medline, Cumulative Index to Nursing and Allied Health Literature and Turning Research Into Practice databases was conducted for relevant guidelines published in the 10 years prior to 31 December 2019. Recommendations surrounding the frequency of testing, lipid-lowering therapies and target cholesterol values were compared qualitatively. Each guideline was assessed using the 2009 Appraisal of Guidelines for Research and Evaluation II tool.

**Results:**

Twenty-two guidelines were included. All recommended statins as the primary lipid-lowering therapy, with a high level of supporting evidence. Considerable variation was found in the recommendations for cholesterol targets. Seventeen guidelines provided at least one cholesterol target, which for low-density lipoprotein (LDL) cholesterol ranged between 1.0 and 2.6 mmol/L, although the most frequently recommended was <1.8 mmol/L (n=12). For long-term follow-up, many recommended reviewing patients annually (n=9), although there was some variation in recommendations for the interval of between 3 and 12 months. Supporting evidence for any approach was limited, often being derived from clinical opinion.

**Conclusions:**

Further research is required to provide an evidence base for optimal lipid monitoring of the on-statin secondary prevention population.

**Key questions**

**What is already known about this subject?**

- There is a large evidence base, supported by the WHO, for the use of statins as lipid-lowering therapy for the prevention of further cardiovascular events. There is no similar guidance regarding the use of cholesterol targets or long-term follow-up in secondary prevention populations.

**What does this study add?**

- A formal, systematic comparison of recommendations of target plasma lipid levels and the frequency of lipid monitoring in the secondary prevention population from 22 guidelines.

**How might this impact on clinical practice?**

- Patients generally prefer to minimise visits to general practitioners, and unnecessary visits and lipid testing also may increase healthcare costs. This work highlights the need for further research to provide an evidence base for optimal lipid monitoring of the secondary prevention population.

## Introduction

In 2016, cardiovascular disease (CVD) was the leading cause of death worldwide and was responsible for an estimated 17.9 million deaths, with heart attacks and strokes accounting for 85% of these.^1^ This is broadly similar to the Global Burden of Disease study’s estimate for 2015 of 17.92 million deaths, which additionally estimated that the number of cases that year was 422.7 million.^2^ With the cardiovascular death rate falling between 1990 and 2015 in most high-income countries,^2^ there is an increasing focus on the management and risk prevention of CVD in the secondary prevention setting.

The need for risk management in secondary prevention, which encompasses coronary heart disease, stroke and peripheral artery disease, is clear. The rate of further cardiovascular events per annum in unmedicated patients with previous events has been estimated to be around 5.6% and 3.7% depending on whether the previous event was coronary heart disease related, compared with 1.8% in those without.^3^ The mortality rate is also six times higher in this population.^4^ As a result of this elevated risk, the WHO states that individuals with established CVD should be treated with lipid-lowering therapy, aspirin, beta-blockers and ACE inhibitors, as well as engage in smoking cessation to reduce the risk of further events by up to 75%.^1^ This has led to the use of statins as lipid-lowering therapy being considered a cornerstone of clinical practice in order to manage secondary CVD risk throughout the world, due to their relative safety, cost, efficacy in lowering cholesterol and consequently CVD prevention. Additionally, there is no threshold beyond which cholesterol lowering is considered dangerous.^3^

However, the WHO has offered no specific guidance regarding target plasma lipid levels or the monitoring of these since the publication of their prevention of cardiovascular guidelines in 2007.^5^ Indeed, different national or international guidelines have contrasting recommendations. For example, the use of on-treatment cholesterol targets has proved controversial in recent years.^6 7^ Prescriptions of higher doses of lipid-lowering therapy are more likely in the pursuit of increasingly lower lipid targets with the aim of reducing a patient’s risk of further cardiovascular events.^8^ However, higher doses of medication also lead to an increased likelihood of side effects,^9 10^ which could result in further costs or even non-adherence or discontinuation in patients, reducing the potential reduction in risk. The long-term follow-up of lipids in high-risk populations poses a significant burden of time to patients,^11^ and costs to healthcare. Specifically, increased biochemistry costs from expanding clinical demand have been flagged as a major financial burden.^12^ Therefore, effectively balancing the costs of follow-up with the reduction in cardiovascular risk within a given population represents a significant challenge, with countries and regions likely to have differing approaches.

This systematic review aimed to investigate similarities and differences in clinical guidelines surrounding the recommendations for the therapeutic treatment, targets and monitoring of lipid risk factors in adults who have established CVD. This will help to highlight variation in the guidelines, thereby providing guidance for future research priorities.

## Methods

A protocol which documented the prespecified analysis and the inclusion criteria for this systematic review was first registered on the International Prospective Register of Systematic Reviews (https://www.crd.york.ac.uk/prospero/) on 19 June 2018 (Ref: CRD42018098582).^13^

### Literature search

A search of Medline, Cumulative Index to Nursing and Allied Health Literature and Turning Research Into Practice databases was conducted for all guidelines published in the ten years prior to 31 December 2019. In addition, several guideline specific databases were searched: National Guideline Clearinghouse (USA), the National Library for Health Guidelines Finder (UK), the Canadian Medical Association Clinical Practice Guidelines Infobase and Guidelines International Network International Guideline Library. Finally, an additional hand search was performed to identify the most recent versions of the guidelines identified through the systematic search. A copy of the search strategy is included in the online supplemental material.

10.1136/openhrt-2020-001396.supp1 Supplementary data

### Selection process

Papers were retained if they met the Institute of Medicine’s 2011 definition of a clinical guideline, ‘Clinical Practice Guidelines are statement that include recommendations intended to optimize patient care that are informed by a systematic review of evidence and an assessment of the benefits and harms of alternative care options’.^14^ As the focus of this systematic review was the management of patients with established CVD, guidelines were only retained within the review if their specific management was detailed, regardless of whether they covered established CVD as a whole, or for the management of patients after a specific event, such as myocardial infarction or stroke. Only the most recent version of the guidelines was retained, with any previous versions removed. Finally, included guidelines had to apply to Organisation for Economic Co-operation and Development countries, produced by a professional organisation, and have the full version of the guidelines available in English.

Two reviewers (REB and JL) independently reviewed the titles and abstracts of the results against the eligibility criteria. The same two reviewers also performed the full-text review, where the reason for exclusion of the guidelines was also documented. In both instances, any discrepancies of opinion were resolved through discussion.

### Data extraction

Data extraction was performed initially by one reviewer (REB) with accuracy checked by a second reviewer (JL). Data extracted included the target population, the publishing society, the country or region the guideline applied to and the year it was published. Recommendations specifically for the secondary prevention population surrounding the frequency that plasma lipid monitoring should be performed, therapies that should be used, and any lipid target values were also extracted if given within the guideline. The strength and the level of evidence of each recommendation were also extracted. Once extracted, the recommendations were compared by all authors.

### Quality assessment

The quality of the development processes of each of these guidelines was then assessed using the 2009 Appraisal of Guidelines for Research and Evaluation (AGREE) II tool by two reviewers (REB and JL). The AGREE II tool consists of 23 questions covering six domains (Scope and Purpose, Stakeholder Involvement, Rigour of Development, Clarity of Presentation, Applicability, and Editorial Independence) and an overall assessment of the quality of the guideline. Each of the items, including the overall quality assessment, is scored on a seven-point scale (1, Strongly Disagree; 7, Strongly Agree), with scaled domain scores then calculated.^15^ As the AGREE II tool does not facilitate an aggregated score across the domains nor a specific cut point for high or low quality, scores for all domains are presented.

## Results

### Results of literature search

**Figure 1:**
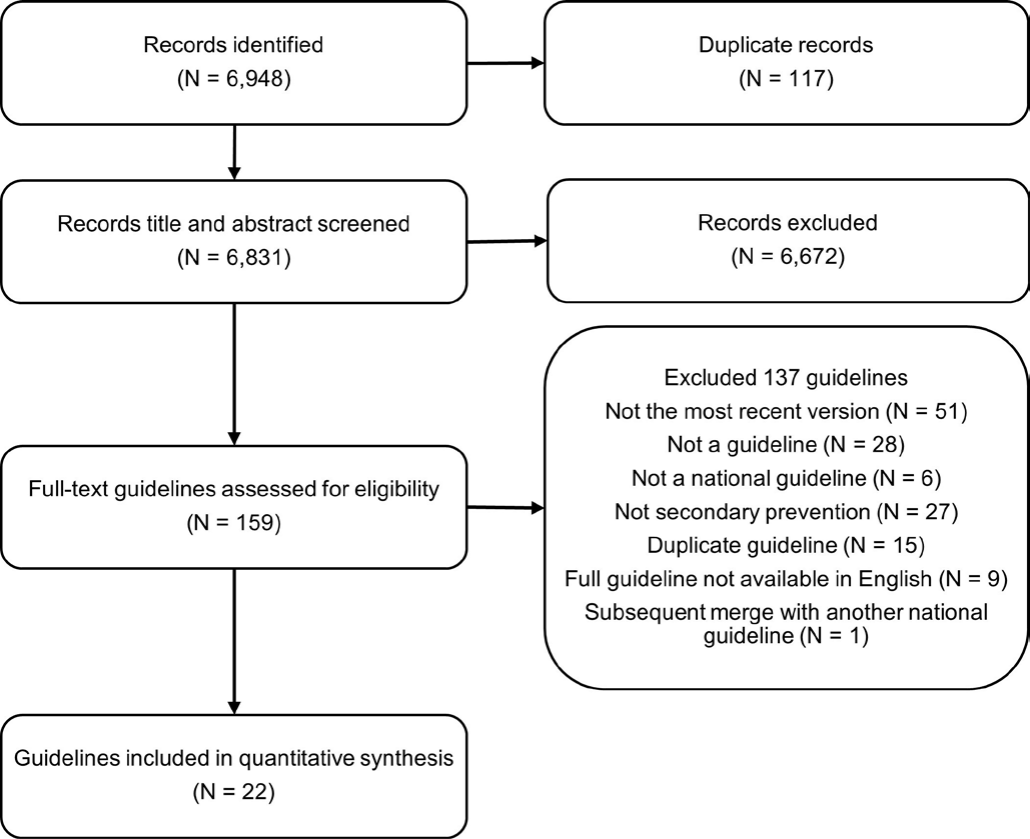
Selection process of relevant guidelines.

The literature search found 6948 results (figure 1), of which 117 were identified as duplicates. Of the 6831 unique results, 6672 were excluded following title and abstract screening. Following a full-text review of the remaining 159 records, a further 137 were excluded. Common reasons for this exclusion were that a more recent version of the guideline existed (n=51), the record was not a guideline (n=28), the guideline did not apply to the secondary prevention population (n=27), or that it was a duplicate copy of another guideline published in a different journal (n=15). The remaining 22 guidelines^16–37^ were assessed for their quality using AGREE II and included in the qualitative comparisons.

### Characteristics and quality of guidelines

The guidelines included are summarised in table 1 and were for 16 different regions. Two of the guidelines were global, with two guidelines each for the USA and Europe. There were also two guidelines each for the UK, South Africa, Australia and New Zealand including one which was applicable to both Australia and New Zealand. Finally, there was one guideline for the following regions: Austria, Canada, China, Hong Kong, Japan, South America, Scotland, Singapore and Taiwan. Most of the guidelines were published in 2014 (n=7), 2016 (n=4), 2017 (n=5) and 2018 (n=4), with one guideline each published in 2010 and 2019.

**Table 1:** Summary of included guidelines

Abbrev.	Development group	Title	Population	Region	Year
ACD	American College of Cardiology, American Heart Association, American Association of Cardiovascular and Pulmonary Rehabilitation, American Association Academy of Physician Assistants, Association of Black Cardiologists, American College of Preventive Medicine, American Diabetes Association, American Geriatrics Society, American Pharmacists Association, American Society for Preventive Cardiology, National Lipid Association, and Preventive Cardiovascular Nurses Association	2018 AHA/ACC/AACVPR/AAPA/ ABC/ACPM/ADA/AGS/APhA/ASPC/NLA/PCNA Guideline on the Management of Blood Cholesterol^16^	All	USA	2018
AUSS	Stroke Foundation	Australian Clinical Guidelines for Stroke Management 2017^30^	Stroke	Australia	2017
AUST	Austrian Obesity Association, Austrian Atherosclerosis Society, Austrian Diabetes Association, Austrian Society of Hypertension, Austrian Society for Internal Angiology, Austrian Society of Nephrology, Austrian Society of Cardiology, Austrian Stroke Society	Austrian Lipid Consensus on the management of metabolic lipid disorders to prevent vascular complications: A joint position statement issued by eight medical societies. 2016 update^23^	All	Austria	2016
CCSG	Canadian Cardiovascular Society	2016 Canadian Cardiovascular Society Guidelines for the Management of Dyslipidemia for the Prevention of Cardiovascular Disease in the Adult^33^	All	Canada	2016
CSN	Chinese Society of Neurology, Cerebrovascular Disease Group	2014 Chinese guidelines for secondary prevention of ischemic stroke and transient ischemic attack^21^	Stroke	China	2014
ESCEAS	European Society of Cardiology, European Atherosclerosis Society	2019 ESC/EAS Guidelines for the management of dyslipidaemias: lipid modification to reduce cardiovascular risk^26^	All	Europe	2019
ESVS	European Society for Vascular Surgery	Management of Atherosclerotic Carotid and Vertebral Artery Disease: 2017 Clinical Practice Guidelines of the European Society for Vascular Surgery (ESVS)^29^	Coronary artery disease	Europe	2017
HKCTF	Hong Kong Cardiovascular Task Force	2016 Consensus statement on prevention of atherosclerotic cardiovascular disease in the Hong Kong population^20^	All	Hong Kong	2016
IAS	International Atherosclerosis Society	An International Atherosclerosis Society Position Paper: global recommendations for the management of dyslipidemia^18^	All	Global	2014
IDF	International Diabetes Federation	Global guideline for type 2 diabetes^19^	Diabetes	Global	2014
JAS	Japan Atherosclerosis Society	Japan Atherosclerosis Society Guidelines for Prevention of Atherosclerotic Cardiovascular Diseases 2017^32^	All	Japan	2017
JBS3	Joint British Societies	Joint British Societies' consensus recommendations for the prevention of cardiovascular disease (JBS3)^37^	All	UK	2014
NHF	National Heart Foundation of Australia, Cardiac Society of Australia and New Zealand	National Heart Foundation of Australia and Cardiac Society of Australia and New Zealand: Australian clinical guidelines for the management of acute coronary syndromes 2016^24^	Acute coronary syndromes	Australia, New Zealand	2016
NLA	National Lipid Association	National Lipid Association recommendations for patient-centered management of dyslipidemia^35^	All	USA	2014
NICE	National Institute for Health and Clinical Excellence	NICE Cardiovascular disease: risk assessment and reduction, including lipid modification^27^	All	UK	2014
NZ	New Zealand Ministry of Health	New Zealand Cardiovascular Disease Risk Assessment and Management for Primary Care^31^	All	New Zealand	2018
SAF	South African Stroke Society	South African guideline for management of ischaemic stroke and transient ischaemic attack 2010^34^	Stroke	South Africa	2010
SAHA	South African Heart Association, Lipid and Atherosclerosis Society of Southern Africa	South African Dyslipidaemia Guideline Consensus Statement: 2018 Update^28^	All	South Africa	2018
SAM	Sociedade Brasileira de Cardiologia	South American guidelines for cardiovascular disease prevention and rehabilitation^36^	All	South America	2014
SIGN	Scottish Intercollegiate Guidelines Network	SIGN 149: Risk estimation and the prevention of cardiovascular disease^25^	All	Scotland	2017
SMH	Singapore Ministry of Health	Singapore Ministry of Health Clinical Practice Guidelines: Lipids^22^	All	Singapore	2017
TSC	Taiwan Society of Cardiology, Taiwan Society of Emergency Medicine, Taiwan Society of Cardiovascular Interventions	2018 Guidelines of the Taiwan Society of Cardiology, Taiwan Society of Emergency Medicine and Taiwan Society of Cardiovascular Interventions for the management of non ST-segment elevation acute coronary syndrome^17^	Acute coronary syndromes (not ST)	Taiwan	2018

Abbrev., abbreviation.

Table 2 contains the AGREE II scores for each of the guidelines. Reflecting generally high quality, eight guidelines were ranked as 4, four ranked as 5, five as 6 and five as 7. Guidelines scored highest in domain 4 (Clarity of Presentation) on average, and lowest in domain 5 (Applicability) with many guidelines scoring below 50%. Scores of 0% were only attained in domains 5 and 6 (Editorial Independence), with the latter occurring when no funding information or conflicts of interest were documented within the text. In terms of maximum values, 100% was attained in all domains by at least one guideline except for domain 3 (Rigour of Development), where the highest score was 96%. This was the largest domain of the six and focused on the development process of the guideline. Many did not document this fully or provide necessary references to additional material, and few detailed their update procedures.

**Table 2:** Appraisal of Guidelines for Research and Evaluation assessment summary scores

Guideline	Domain 1 (%)	Domain 2 (%)	Domain 3 (%)	Domain 4 (%)	Domain 5 (%)	Domain 6 (%)	Overall quality (/7)
ACD	89	50	65	100	63	100	7
AUSS	100	83	90	89	50	100	6
AUST	78	33	27	61	0	33	4
CCSG	78	39	65	94	38	100	6
CSN	56	44	48	78	17	83	4
ESCEAS	56	61	63	94	54	100	7
ESVS	78	56	75	72	13	33	5
HKCTF	50	33	46	78	25	58	4
IAS	44	39	42	44	25	42	4
IDF	56	33	48	83	100	83	6
JAS	61	44	71	67	4	25	4
JBS3	56	56	29	94	25	50	5
NHF	89	50	73	94	79	67	7
NICE	100	78	94	100	96	100	7
NLA	72	61	54	94	17	92	6
NZ	83	39	27	78	17	17	4
SAF	39	56	67	78	29	92	6
SAHA	61	56	31	72	29	83	5
SAM	33	28	29	61	33	33	4
SIGN	89	100	96	89	88	100	7
SMH	67	67	54	94	17	0	4
TSC	67	50	52	94	8	50	5

Domain 1, Scope and Purpose; Domain 2, Stakeholder Involvement; Domain 3, Rigour of Development; Domain 4, Clarity of Presentation; Domain 5, Applicability; Domain 6, Editorial Independence.

ACD, American College of Cardiology/American Heart Association/American Association of Cardiovascular and Pulmonary Rehabilitation/American Association Academy of Physician Assistants/Association of Black Cardiologists/American College of Preventive Medicine/American Diabetes Association/American Geriatrics Society/American Pharmacists Association/American Society for Preventive Cardiology/National Lipid Association/Preventive Cardiovascular Nurses Association; AUSS, Australia Stroke Society; AUST, Austrian Obesity Association/Austrian Atherosclerosis Society/Austrian Diabetes Association/Austrian Society of Hypertension/Austrian Society for Internal Angiology/Austrian Society of Nephrology/Austrian Society of Cardiology/Austrian Stroke Society; CCSG, Canadian Cardiovascular Society; CSN, Chinese Society of Neurology and Cerebrovascular Disease Group; ESCEAS, European Society of Cardiology and European Atherosclerosis Society; ESVS, European Society for Vascular Surgery; HKCTF, Hong Kong Cardiovascular Task Force; IAS, International Atherosclerosis Society; IDF, International Diabetes Federation; JAS, Japan Atherosclerosis Society; JBS3, Joint British Societies; NHF, National Heart Foundation of Australia and Cardiac Society of Australia and New Zealand; NICE, National Institute for Health and Clinical Excellence; NLA, National Lipid Association; NZ, New Zealand Ministry of Health; SAF, South African Stroke Society; SAHA, South African Heart Association; SAM, Sociedade Brasileira de Cardiologia; SIGN, Scottish Intercollegiate Guidelines Network; SMH, Singapore Ministry of Health; TSC, Taiwan Society of Cardiology/Taiwan Society of Emergency Medicine/Taiwan Society of Cardiovascular Interventions.

### Summary of recommendations

Recommendations for the use of statin medication, cholesterol targets and the frequency of monitoring are presented in table 3.

**Table 3:** Summary of recommendations

Guideline	Statin medication			Cholesterol targets			Frequency of monitoring		
	Recommendation	LoE	SoR	Recommendation	LoE	SoR	Recommendation	LoE	SoR
ACD	≤75 year: high-intensity statins >75 year: initiate moderate or high-intensity statins if benefit–risk ratio favourable	A B	I IIa	–	–	–	Fasting lipids 4–12 weeks after initiation, then every 3–12 months	A	I
AUSS	*Atherosclerotic*: high-intensity statins if reasonable life expectancy	High	Strong	–	–	–	–	–	–
AUST	Statins	–	–	LDL-C <1.8 mmol/L Or LDL-C >50% reduction Non-HDL-C<2.6 mmol/L	– – –	– – –	–	–	–
CCSG	Moderate/high-intensity statins	High	Strong	LDL-C <2.0 mmol/L Or LDL-C >50% reduction Alternatively, apoB <0.8 g/L Or non-HDL-C<2.6 mmol/L ACS: LDL-C <1.8 mmol/L Or LDL-C >50% reduction	Mod Mod Mod Mod – –	Strong Strong Strong Strong V&Ps V&Ps	Until stable	–	PO
CSN	Ischaemic: high-intensity statins	A	I	LDL-C <1.8 mmol/L Or LDL-C ≥50% reduction	B B	II II	–	–	–
ESCEAS	Baseline LDL-C >1.4 mmol/L: offer med <1.4 mmol/L: consider med	A A	I IIa	LDL-C <1.4 mmol/L Or LDL-C >50% reduction Further event <2 years: LDL-C <1.0 mmol/L	A A B	I I IIb	Starting/adjusting: 8 (±4) weeks Once achieved: Annually	– –	– –
ESVS	Statins prior to endarterectomy or stenting	A	I	--	–	–	–	–	–
HKCTF	High-intensity statins	–	–	LDL-C <1.8 mmol/L Or LDL-C >50% reduction if baseline 1.8-3.5 mmol/L	– –	– –	–	–	–
IAS	Maximal statins	–	–	LDL-C <1.8 mmol/L. Non-HDL-C<2.6 mmol/L	– –	– –	–	–	–
IDF	Statins	–	RC	Triglyceride <2.3 mmol/L HDL-C >1.0 mmol/L. Non-HDL-C<2.5 mmol/L LDL-C <1.8 mmol/L	– – – –	RC RC RC RC	At least annually	–	RC
JAS	Statins	1+	A	LDL-C <2.6 mmol/L Or LDL-C >50% reduction if target cannot be met If additional conditions: LDL-C <1.8 mmol/L If triglyceride high: Non-HDL-C<3.4 mmol/L Non-HDL-C<2.6 mmol/L if additional conditions	3 3 – – –	A A – – –	Regular blood testing Every 3–6 months	– –	B –
JBS3	Atorvastatin, up to 80 mg in ACS	–	–	LDL-C <2.0 mmol/L Non-HDL-C<2.5 mmol/L	– –	– –	Annual non–fasting TC and HDL–C once stable	–	–
NHF	Highest tolerated dose of statins	1A	Strong	LDL-C ≤1.8 mmol/L	–	–	TC and LDL–C approx. 3 months after starting	–	–
NICE	Atorvastatin 80 mg, lower dose if not tolerated.	–	Strong	Non-HDL-C>40% reduction	–	Strong	3 months after treatment start. Annual non–fasting lipids	– –	Strong Weak
NLA	Moderate/high-intensity statins	High	A	Non-HDL-C<2.6 mmol/L LDL-C <1.8 mmol/L	High High	A A	4–12 months once achieved	Low	E
NZ	Statins	–	–	LDL-C 1.6-1.8 mmol/L	–	–	Non–fasting 6–12 months until target achieved. Annually.	– –	– –
SAF	Atherosclerotic and TC >3.5 mmol/L: statins Trial strength, for example, 40 mg simvastatin	I –	A –	–	–	–	–	–	–
SAHA	High-intensity statins	–	–	LDL-C <1.8 mmol/L Or LDL-C >50% reduction if baseline 1.8-3.5 mmol/L	– –	– –	Starting/adjusting: 8 (±4) weeks Once achieved: 6 months	– –	– –
SAM	Statins	–	–	LDL-C <2.6 mmol/L	–	–	–	–	–
SIGN	Atorvastatin 80 mg Lower if not tolerated	– –	Strong GP	–	–	–	Annual Review	–	GP
SMH	Statins	1++	A	LDL-C <2.1 mmol/L	1++	A	Annually	–	GP
TSC	Statins	A	I	LDL-C <1.8 mmol/L Diabetes: LDL-C <1.4 mmol/L	B B	I IIa	–	–	–

ACD, American College of Cardiology/American Heart Association/American Association of Cardiovascular and Pulmonary Rehabilitation/American Association Academy of Physician Assistants/Association of Black Cardiologists/American College of Preventive Medicine/American Diabetes Association/American Geriatrics Society/American Pharmacists Association/American Society for Preventive Cardiology/National Lipid Association/Preventive Cardiovascular Nurses Association; ACS, acute coronary syndromes; AUSS, Australia Stroke Society; AUST, Austrian Obesity Association/Austrian Atherosclerosis Society/Austrian Diabetes Association/Austrian Society of Hypertension/Austrian Society for Internal Angiology/Austrian Society of Nephrology/Austrian Society of Cardiology/Austrian Stroke Society; CCSG, Canadian Cardiovascular Society; CSN, Chinese Society of Neurology and Cerebrovascular Disease Group; ESCEAS, European Society of Cardiology and European Atherosclerosis Society; ESVS, European Society for Vascular Surgery; GP, good practice; (non) HDL-C, (non) HDL-cholesterol; HKCTF, Hong Kong Cardiovascular Task Force; IAS, International Atherosclerosis Society; IDF, International Diabetes Federation; JAS, Japan Atherosclerosis Society; JBS3, Joint British Societies; LDL-C, LDL cholesterol; LoE, level of evidence; Med, medication; Mod, moderate; NHF, National Heart Foundation of Australia and Cardiac Society of Australia and New Zealand; NICE, National Institute for Health and Clinical Excellence; NLA, National Lipid Association; NZ, New Zealand Ministry of Health; PO, panel opinion; RC, recommended care; SAF, South African Stroke Society; SAHA, South African Heart Association; SAM, Sociedade Brasileira de Cardiologia; SIGN, Scottish Intercollegiate Guidelines Network; SMH, Singapore Ministry of Health; SoR, strength of recommendation; TC, total cholesterol; TSC, Taiwan Society of Cardiology/Taiwan Society of Emergency Medicine/Taiwan Society of Cardiovascular Interventions; V&Ps, values and preferences.

### Treatment recommendations

All of the guidelines presented recommendations for the treatment of the secondary prevention population, with all recommending statins as the primary therapy. Only UK/Scottish guidelines suggested the specific drug and dose, namely, atorvastatin 80 mg, with many instead recommending the maximally tolerated high-intensity doses in general, with lower doses considered when contraindications were present, or they were poorly tolerated by the patients. Few caveats were stated regarding the prescription of statins. For example, all stroke guidelines recommended statins only when the cause of the stroke was likely to be atherosclerotic, with the South African Stroke Society additionally only recommending them in the case of total cholesterol >3.5 mmol/L, and the Australia Stroke Society only considering them appropriate when the patient’s life expectancy was considered reasonable. For the secondary prevention population as a whole, the European Society of Cardiology and European Atherosclerosis Society (ESCEAS) tailored their recommendations for patients whose baseline LDL was <1.4 mmol/L at baseline, respectively, with therapy considered rather than offered to these patients. Finally, the 2018 American Consensus (ACD) guidelines stated that the benefit–risk ratio should be considered when offering medication to patients over the age of 75 years.

For the guidelines which reported corresponding levels of evidence with their treatment recommendations (n=12), all considered that the level of evidence for statins was high, resulting in strong recommendations for their administration to the secondary prevention population. For situations where lower doses of therapy may be needed, such as in cases of contraindications or lack of tolerance, if specified at all, guidelines often considered the level of evidence supporting these changes to be lower than for the main treatment recommendation. Specifically, the level of evidence was typically assessed to be moderate (rather than high) or such alterations to medications were considered to be only good practice.

Besides statins, other lipid-lowering medications were also discussed within the guidelines (online supplemental table S1). The most commonly recommended of which was ezetimibe (n=17), both as an additional medication (n=15), and as a monotherapy (n=10) predominantly for patients with statin intolerance (n=8). Fibrates, niacin derivatives and omega-3 supplements were also commonly recommended (n=15, n=10 and n=8, respectively) though under two different circumstances: elevated triglyceride levels and LDL cholesterol lowering. For the former, 14 recommended fibrates, while 5 guidelines each recommended considering niacin derivatives and Omega-3 supplements. Three guidelines suggested Omega-3 as lipid-lowering therapy, although the roles of fibrates and niacin derivatives were more disputed. Fibrates and niacin derivatives were recommended routinely in five and eight guidelines, respectively. However, three guidelines each did not recommend the use of fibrates and niacin derivatives. Bile acid sequestrant use was debated in 13 guidelines, with only the South African Heart Association discouraging their use. Proprotein Convertase Subtilisin/Kexin type 9 (PCSK9) inhibitors were only included in six guidelines, all of which were published from 2016 onwards, and all recommending them as an additional therapy or in cases of statin intolerance. Four guidelines did not give any recommendations for lipid-lowering medications other than statins. Evidence supporting these recommendations (if stated) was generally assessed by the guidelines to be of lower quality than for statins, and consequently the associated strength of recommendations was typically lower. Ezetimibe and PCSK9 inhibitors tended to have higher levels of supporting evidence behind them, although the strength of recommendations for PCSK9 inhibitors was lower due to their recent approval and limited long term follow-up of cardiovascular events.

### Plasma lipid recommendations

Seventeen of the guidelines provided at least one target, with all except one of these providing an LDL cholesterol goal. Target values ranged between 1.0 and 2.6 mmol/L, although the most frequently recommended was <1.8 mmol/L (n=12). Many guidelines additionally suggested that a 50% reduction in LDL could be used as an alternative where this target may be unattainable or for patients whose baseline values were already <3.5 mmol/L (n=7). A non-high-density lipoprotein (HDL) cholesterol target was also common (n=8), with target values ranging from 2.5 to 3.4 mmol/L, with 2.6 mmol/L the most frequent (n=5). These targets were usually given in combination with a target for LDL cholesterol, though in the case of the Japan Atherosclerosis Society, the non-HDL target was considered only relevant when a patient’s triglycerides were elevated. Meanwhile, the National Institute for Health and Clinical Excellence did not provide a numerical target for non-HDL, recommending a 40% reduction from the patient’s baseline only. Only two guidelines referred to other lipid parameters in their recommendations. The Canadian Cardiovascular Society (CCSG) provided an apolipoprotein B target as an alternative for LDL, and the International Diabetes Federation provided additional targets for triglycerides and HDL cholesterol. There were no apparent differences in recommendations for stroke or diabetes-specific guidelines, although some guidelines for all secondary prevention populations provided different targets for those patients with additional comorbidities (n=2), or specific cardiovascular events (n=2).

For the majority of the guidelines that provided targets, the recommendations provided either no supporting evidence or graded it as low. Consequently, the associated strength of the recommendations was often either not given or was given as preferences and opinions of those involved in the guideline’s construction. There were few exceptions. Singapore’s (SMH) recommendation of LDL <2.1 mmol/L considered the level of evidence to be high, resulting in a strong recommendation. The target of LDL <1.8 mmol/L, when stated in some guidelines, was also strongly recommended, with the National Lipid Association considering the supporting evidence to be high, while others graded it as only moderate (n=2). CCSG’s target for patients who have not experienced an acute coronary syndrome of LDL <2.0 mmol/L was also given as a strong recommendation, with the guideline viewing the supporting evidence for it as moderate. Its recommendation of LDL <1.8 mmol/L for patients who have experienced an acute coronary syndrome cited no supporting evidence, and consequently was given as a preference among the guideline’s creators.

### Frequency of monitoring

Thirteen guidelines detailed recommendations regarding the ongoing monitoring of this population, with the specifics falling into three categories: monitoring following the initiation of treatment (n=5), monitoring prior to stable lipids (n=2), and long-term follow-up (n=11), with some providing recommendations in more than one of these categories (n=5). Of those who detailed monitoring following statin initiation, all recommended a review of the patient’s lipids within 3 months. Within these, two guidelines recommended also measuring the patient’s alanine aminotransferase at this review,^26 27^ one suggested this should be conducted only if symptoms were present,^16^ and another two did not refer to this safety blood indicator.^24 28^ Furthermore, the measurement of creatine kinase was only considered where side effects were reported at this initial review, and was recommended in five of these guidelines,^16 26–28^ and implied in the remaining guideline.^24^ Meanwhile, for the guidelines which recommended monitoring prior to stability, the criteria for this were not clearly defined. Both CCSG and New Zealand Ministry of Health (NZ) did not detail a specific purpose for these reviews. In terms of long term follow-up, many recommended reviewing the patient annually (n=9), although there was some variation in recommendations between 3 and 12 months.

The majority of guidelines considered the evidence behind their frequency of monitoring recommendations to either be low (n=1) or gave no evidence to support them (n=11), sometimes referring to them as good practice points or clinician’s opinions or preferences (n=5). However, the ACD guidelines were the exception to this, which graded the evidence behind their monitoring recommendations of every 3–12 months as strong.

## Discussion

This systematic review illustrates the variation in recommendations surrounding optimal on-statin lipid monitoring within secondary prevention. Specifically, there were considerable differences in the recommendations for cholesterol targets (including their use) and the ongoing monitoring of lipid levels over the longer term. These findings reflect the fact that no guideline identified a specifically designed randomised controlled trial to assess either treatment targets or monitoring of therapy. However, such trials are likely to be expensive, although in the future advances with electronic health records may facilitate the evidence base for this. Nonetheless, this systematic review illustrates that better evidence is needed to provide an optimal approach to lipid monitoring in order to balance safety, adherence, cost, and time burden to patients.

All guideline committees are likely to be searching a broadly similar evidence base, where the efficacy and safety of statins has been well established in the prevention of further cardiovascular events.^3 38^ Furthermore, the WHO recommends the use of statins as part of their secondary prevention programme,^1^ with the increased risk widely accepted within this population. It is therefore not surprising that all guidelines agree that statins should be commenced as the lipid-lowering therapy with a high level of evidence, commensurate with the availability of randomised controlled trials. Guidelines that specify drugs or doses generally recommend high dose therapy and titrating down as necessary to a tolerated dose, rather than titrating up. This is in line with large randomised controlled trials over the preceding decades that have shown that higher dose statin therapy improves outcomes.^10^ Nonetheless, despite this widespread recommendation, there is some evidence to suggest that statins are not consistently prescribed within the secondary prevention population depending on the cardiovascular events experienced.^39^ Meanwhile, recommendations for the use of other lipid-lowering therapies illustrate that such guidelines are likely a reflection of the evidence available when the guidelines were created; for example, PCSK9 inhibitors were only discussed in guidelines published after 2016. Therefore, in clinical practice, consideration may need to be given to the timing of the publication where new evidence has emerged.

The evidence that ‘lower is better’ for LDL and non-HDL cholesterol in terms of secondary CVD prevention is well supported by evidence, and the recent advent of PCSK9 inhibitors further supports this notion.^40 41^ The issue at hand is how to use this information clinically to support a testing regimen. In this regard, the use of cholesterol targets for therapy is contentious, in part because the evidence for their use is less strong. While research has shown that achieving targets is associated with better outcomes,^40–42^ no specific randomised controlled trial has shown that randomising patients to a target improves adherence or event rates. Indeed, it might be argued that the maximally tolerated statin should be initiated as the default, and therefore a hard target may be moot. Clearly, other lipid-lowering medications could be added to therapy. Despite the lack of strong evidence, many guidelines recommended specific lipid targets, with many choosing similar values, suggesting that guideline committees are likely to be examining the same evidence. However, one of the most recently published guidelines, the 2019 ESC/EAS Guidelines for the Management of Dyslipidaemias (ESCEAS) advised the lowest cholesterol targets of all the included guidelines (LDL <1.4 mmol/L), with further lower levels for those with multiple recent cardiovascular events (LDL <1.0 mmol/L). This was rated as being supported by strong and moderate levels of evidence, although the guidelines acknowledged that both targets are based on the LDL levels achieved in the trials for PCSK9 inhibitors.^26 40 41^

There was virtually no evidence to support any recommendations regarding the frequency of ongoing monitoring once lipid-lowering therapies had been commenced. Guidelines that recommended retesting following statin initiation tended to additionally recommend liver function tests were performed as a safety indicator, especially if hepatic symptoms were present, including those produced by the ACC/AHA and the ESC/EAS.^16 26^ Guidelines frequently conflate the issue of short term safety bloods with longer term lipid monitoring when reporting the strength of evidence. Despite this, only one guideline (ACD) cited evidence that they considered to be strong in their recommendations. The evidence referenced would suggest that monitoring patients regularly is associated with improved adherence to medication, and, consequently, patient outcomes.^43^ However, this study was open to confounding due to its observational nature, and as far as we are aware, this has never been tested within a randomised controlled trial. Furthermore, the purpose of such follow-up testing in guidelines is seldom stated. Where evidence was cited, though, this would suggest that the purpose of such reviews is to promote adherence, but this may not be the rationale for all of the guidelines included, which could also include the monitoring of lipids to check if targets are still being achieved or of safety concerns. Regardless, the majority of guidelines recommended that secondary prevention patients were reviewed annually based purely on clinician’s opinion. However, in the UK, simulations have suggested that this is likely to be optimal economically as well as reducing the impact of any natural variation in an individual’s cholesterol levels.^44^ Nonetheless, some patients will not be optimally managed under these recommendations, and by integrating algorithms into electronic health records to aid clinical decision making, there is the potential to personalise an individual patient’s lipid management.

### Strengths and limitations

To our knowledge, this is the first review to compare guidelines surrounding the management of lipids in the secondary prevention population, as previous research has focused on comparing guidelines for assessing risk and managing it through lifestyle interventions in the primary prevention population only.^45 46^ Furthermore, following a comprehensive search, this identified current guidelines from 22 different professional bodies, covering 16 different geographical regions. Nonetheless, guidelines were only included if their full guideline was available in English, which is likely to have resulted in a bias in the regions included and impacted the number of guidelines compared within this review. Another limitation is that the guidelines’ methodological quality, as assessed by the AGREE II tool, was not used to restrict their inclusion in this review, and no comparisons were made between either the recommendations given, their considered level of evidence or their strength, and the AGREE scores. However, given that all included guidelines were assessed to be of generally high quality, such stratification is unlikely to have yielded meaningful differences.

## Conclusion

The safe and optimal treatment of plasma lipids within the secondary prevention population is key to reducing the increasing burden of CVD in society. However, given the paucity of evidence for the frequency of ongoing monitoring, there is a clear need for further research in these two key areas of its management. This will improve patient care while optimising costs in an evidence-based manner.
