# Supplementary material for: Systematic review of clinical guidelines for lipid lowering in the secondary prevention of cardiovascular disease events
Source: Open Heart. 2020 Dec 18;7(2):e001396. doi: 10.1136/openhrt-2020-001396 (PMC7751215; doi:10.1136/openhrt-2020-001396)
Supplement: Supplementary data [file openhrt-2020-001396supp001.pdf]

BMJ Publishing Group Limited (BMJ) disclaims all liability and responsibility arising from any reliance placed on this supplemental material which has been supplied by the author(s)

Supplementary Material

Example Search Strategy

((Cardiovascular Diseases/)

OR (exp Aortic Aneurysm/)

OR (exp Myocardial Ischemia/)

OR (exp ARTERIOSCLEROSIS/)

OR (exp Cerebrovascular Disorders/)

OR (Peripheral Vascular Diseases/)

OR (exp Heart Failure/)

OR ((cardiovascular adj3 disease*).mp.)

OR ((coronary adj3 disease*).mp.)

OR (heart disease*.mp.)

OR ((stroke* or cerebrovasc* or cva*).mp.)

OR ((aort* adj5 aneurysm).mp.)

OR ((abdominal adj5 aneurysm).mp.)

OR ((thoracoabdominal adj5 aneurysm).mp.)

OR ((arteri* adj3 occlusi*).mp.)

OR ((arteri* adj3 stenosis).mp.)

OR ((peripher* adj5 occlusi*).mp.)

OR ((peripher* adj5 arteri*).mp.)

OR ((peripher* adj5 vascular).mp.)

OR (heart failure.mp.)

OR (atherosclerosis.mp.)

OR (arteriosclerosis.mp.)

OR (HYPERTENSION/)

OR (Hyperlipidemias/)

OR (Diabetes Mellitus/)

OR (hypertension.mp.)

OR (hyperlipid?emia.mp.)

OR (dyslipid?emia.mp.)

OR (cholesterol.mp.)

OR (diabetes.mp.)

OR (metabolic syndrome.mp.))

AND

((Cardiovascular Diseases/pc [Prevention & Control])

OR (Secondary Prevention/)

OR (Risk Assessment/)

OR ((established adj3 disease).mp.)

OR ((subsequent adj3 event*).mp.)

OR ((recurrent adj3 event*).mp.)

OR ((multiple adj3 event*).mp.)

OR ((secondary adj3 prevention).mp.)

OR ((subsequent adj3 episode*).mp.)

OR ((recurrent adj3 episode*).mp.)

OR ((prior adj3 cardiovascular).mp.)

OR ((previous adj3 cardiovascular).mp.)

OR ((multiple adj3 episode*).mp.))

AND

((Critical Pathways/)

OR (exp Clinical Protocols/)

OR (exp CONSENSUS/)

OR (exp Consensus Development Conference/)

OR (exp Consensus Development Conferences as Topic/)

OR (exp GUIDELINE/)

OR (Guidelines as Topic/)

OR (exp Practice Guideline/)

OR (Practice Guidelines as Topic/)

OR (Health Planning Guidelines/)

OR ((guideline or practice guideline or consensus development conference or consensus development conference, NIH).pt.)

OR ((position statement* or policy statement* or practice parameter* or best practice*).ti,ab,kf,kw. (standards or guideline or guidelines).ti,kf,kw.)

OR (((practice or treatment* or clinical) adj guideline*).ab.)

OR ((CPG or CPGs).ti.)

OR (consensus*.ti,kf,kw.)

OR (consensus*.ab. /freq=2)

OR (((critical or clinical or practice) adj2 (path or paths or pathway or pathways or protocol*)).ti,ab,kf,kw.)

OR (recommendat*.ti,kf,kw.)

OR ((care adj2 (standard or path or paths or pathway or pathways or map or maps or plan or plans)).ti,ab,kf,kw.)

OR ((algorithm* adj2 (screening or examination or test or tested or testing or assessment* or diagnosis or diagnoses or diagnosed or diagnosing)).ti,ab,kf,kw.)
OR ((algorithm* adj2 (pharmacotherap* or chemotherap* or chemotreatment* or therap* or treatment* or intervention*)).ti,ab,kf,kw.))

NOT

((comment.pt.)
OR (letter.pt.)
OR (editorial.pt.))

Limit results to English language
Limit results to yr="2010 -Current"

Table S1: Summary of Non-Statin Medication Recommendations

	Ezetimibe			Fibrates			PCSK9 Inhibitors			Others		
**Guideline**	**Recom.**	**LoE**	**SoR**	**Recom.**	**LoE**	**SoR**	**Recom.**	**LoE**	**SoR**	**Recom.**	**LoE**	**SoR**
ACD	If max statins and LDL ≥1.8 mmol/l, +	B-R	IIa	If trigs ≥5.7 mmol/l	B -NR	IIa	If max LLM, non-HDL ≥2.6 or LDL ≥1.8 mmol/l, +	A	IIa	--	--	--
AUSS	--	--	--	Not routine	Mod	Weak	--	--	--	--	--	--
AUST	First-line option	--	--	If trigs ≥5.6 mmol/l<br>For lowering LDL, 2nd choice	--<br>--	--<br>--	--	--	--	For lowering LDL,<br>BAS<br>Niacin 2nd choice<br>If trigs ≥5.6 mmol/l<br>Niacin<br>Omega-3 3-4g	 --<br>--<br><br>--<br>--	 --<br>--<br><br>--<br>--
CCSG	If max statins, +	High	Strong	For lowering LDL once met, + not recommended<br>If high trigs, low HDL, may benefit	High<br>--	Strong<br>V&Ps	If max statins (/+ezetimibe), consider +	Mod	Cond	For lowering LDL once met, + niacin not recommended<br>If max statins (/+ezetimibe), consider + BAS	High<br>Low	Strong<br>Cond

CSN	--	--	--	--	--	--	--	--	--	--	--	--
ESCEAS	If max statins and LDL above target, +<br>If statin intolerant, consider	B<br>C	I<br>IIa	If trigs >2.3 mmol/l and LDL at target, consider + feno- or bezafibrate	C	IIa	If max statin + ezetimibe, +<br>If statin intolerant, consider + ezetimibe	A<br>C	I<br>IIb	If max statins, consider + BAS<br>If trigs 1.5-5.6 mmol/l, consider + Omega-3	C<br>B	IIb<br>IIa
ESVS	--	--	--	--	--	--	--	--	--	--	--	--
HKCTF	If max statins, +<br>If statin intolerant	--<br>--	--<br>--	If very high trigs	--	--	--	--	--	If low HDL or high trigs, + nicotinic acid<br>If statin intolerant:<br>BAS<br>Nicotinic acid	--<br><br>--<br>--	--<br><br>--<br>--
IAS	If max statins, LDL ≥ 1.8mmol/l, consider +	--	--	If high trigs, low HDL, target LDL, consider +	--	--	--	--	--	If max statins, LDL ≥1.8 mmol/l, consider + BAS<br>If high trigs, low HDL, target LDL, consider +:<br>Niacin	--<br><br>--	--<br><br>--

										High dose Omega-3	--	--
IDF	If max statins,	--	RC	If trigs > 2.3mmol/l,	--	RC	--	--	--	<u>If max statins,</u>		
	consider +			low HDL, consider						<u>consider +:</u>		
	If statin intolerant	--	RC	fenofibrate						BAS	--	RC
										Nicotinic Acid	--	RC
										Omega-3	--	RC
										<u>If statin intolerant:</u>		
										BAS	--	RC
										Nicotinic Acid	--	RC
										Omega-3	--	RC
JAS	+ with statins	1+	B	Monotherapy	--	--	+ with statins	1+	B	BAS	--	--
	Monotherapy	--	--	If high trigs, consider	--	--				Omega-3	--	--
				+						Nicotinic Acid	--	--
JBS3	If max statins,	--	--	If trigs >10mmol/l	--	--	--	--	--	If max statins,	--	--
	consider + 10mg									consider BAS		
	If statin intolerant,	--	--									
	consider 10mg											
NHF	If max statins,	--	--	--	--	--	--	--	--	--	--	--
	consider + 10mg											
		--	--									

	If statin intolerant,											
	consider 10mg											
NICE	--	--	--	--	--	--	--	--	--	--	--	--
NLA	If max statins,	Mod	A	If max statins,	Mod	A	--	--	--	<u>If max statins,</u>		
	consider +			consider +						<u>consider +:</u>		
	If statin intolerant	High	A	If statin intolerant	High	A				BAS	Mod	A
										Omega-3	Mod	A
										Nicotinic Acid	Mod	A
										<u>If statin intolerance</u>		
										BAS	High	A
										Omega-3	High	A
										Nicotinic Acid	High	A
NZ	If statin intolerant,	--	--	If high trigs	--	--	--	--	--	If statin intolerant,	--	--
	consider			If statin intolerant,	--	--				consider niacin		
				consider								
SAF	--	--	--	--	--	--	--	--	--	--	--	--
SAHA	If max statins,	--	--	If trigs >2.3 mmol/l,	--	--	If max statins,	--	--	<u>LDL lowering:</u>		
	consider +			fenofibrate			consider +			BAS discouraged	--	--
										Nicotinic acid	--	--
										discouraged		

										If trigs >2.3 mmol/l:<br>+ Omega-3 (2-4g)<br>+ Nicotinic Acid	 --<br>--	 --<br>--
SAM	If max statins, +	--	--	If max statins, +<br>If trigs >5.6 mmol/l, +	--<br>C	--<br>I	--	--	--	If low HDL, + niacin<br>If max statins:<br>+ BAS<br>+ Niacin	--<br><br>--<br>--	--<br><br>--<br>--
SIGN	If max statins, consider +	--	Strong	For lipid lowering, not routine<br>If trigs high, low HDL, consider	--<br>--	Strong<br>GP	If max LLM, consider	--	Strong	If max statins, consider + BAS<br>Nicotinic acid not recommended	--<br>--	Strong<br>Strong
SMH	If max statins, +<br>If statin intolerant	1++<br>1++	A<br>A	If low HDL, trigs 2.3-4.5 mmol/l, consider +<br>Prefer fenofibrate<br>If trigs >10 mmol/l	2+<br>3<br>1+	C<br>D<br>A	--	--	--	If max statins, + BAS<br>If max statins, consider + niacin<br>If trigs ≥4.5 mmol/l, consider niacin<br>If trigs >10 mmol/l, + Omega-3	4<br>1+<br>1+<br>1+	D<br>A<br>A<br>A
TSC	+ with statin	A	I	--	--	--	--	--	--	--	--	--

BMJ Publishing Group Limited (BMJ) disclaims all liability and responsibility arising from any reliance placed on this supplemental material which has been supplied by the author(s)

PCSK9, Proprotein Convertase Subtilisin/Kexin type 9; LoE, Level of Evidence; SoR, Strength of Recommendation; +, Adding; Trigs, Triglycerides; LLM, Lipid Lowering Medication; Mod, Moderate; BAS, Bile Acid Sequestrant; V&Ps, Values and Preferences; Cond, Conditional; RC, Recommended Care; GP, Good Practice;
